# Exploring the factors influencing adherence to oral anticancer drugs in patients with digestive cancer: a qualitative study

**DOI:** 10.1007/s00520-021-06663-2

**Published:** 2021-11-23

**Authors:** Pierre Nizet, Yann Touchefeu, Solange Pecout, Estelle Cauchin, Eva Beaudouin, Séverine Mayol, Clémentine Fronteau, Jean-François Huon

**Affiliations:** 1 Clinical Pharmacy Unit, Nantes University Hospital, 1 Rue Gaston Veil, 44000 Nantes, France; 2 Digestive Oncology, Institut Des Maladies De L’Appareil Digestif, Nantes University Hospital, Nantes, France; 3 INSERM, UMR 1246—SPHERE, MethodS in Patients-Centered Outcomes and HEalth ResEarch, Nantes and Tours, France; 4 Research Department, Nantes University Hospital, Nantes, France

**Keywords:** Adherence, Oral anticancer drugs, Qualitative research, Patient experience

## Abstract

**Purpose:**

The aim of this study was to explore the beliefs, perceptions and representations of patients in order to identify the determinants of oral anticancer drugs adherence and to take action in current practice to improve patient support in digestive oncology.

**Methods:**

We constructed a semi-directed interview guide which aimed to explore the patient’s relationship with medication, their health history, their experiences at the time of the announcement of treatment, their confidence, their fears, their motivations to adhere to their treatment and the constraints linked to their treatment. The data were analysed and discussed using a thematic approach.

**Results:**

Seventeen patients agreed to participate in the study. The median age was 60 years. Ten patients had colorectal cancer, 3 patients had hepatocellular carcinoma, 3 patients had gastrointestinal stromal tumour and 1 patient had neuroendocrine pancreatic tumour. We identified five categories of factors influencing adherence: demographic and socioeconomic, disease-related, treatment-related, care system-related, and patient representation and pathways’ factors. A majority of patients emphasised the importance of family support in the adherence process and the convenience of per os treatment compared to other intravenous treatments. However, several negative determinants emerged such as the toxicity of the treatment, fears of forgetting to take the medication, difficulties with the galenic formulation and negative beliefs of the family.

**Conclusion:**

This study demonstrates the need to address the different dimensions of the patient in order to understand his or her behaviour with regard to adherence and to identify the levers for improvement.

**Supplementary Information:**

The online version contains supplementary material available at 10.1007/s00520-021-06663-2.

## Introduction

Compliance is defined as “the extent to which an individual’s behaviours (in terms of taking medication, following a diet or changing lifestyle) coincide with medical or health advice” [1]. The concept of compliance can be summarised as a relationship between “what the patient does” and “what the doctor says” [2]. More concretely, it can be defined as a ratio between the actual number of drugs taken by the patient over a given period and the total number of drugs prescribed by the doctor over the same period. This notion of compliance is increasingly assimilated to an attitude of obedience on the part of the patient towards the carer [3]. Therapeutic adherence or adherence to treatment is defined as the patient’s considered approval of the management of his or her illness and treatment, and therefore as the patient’s acceptance of the treatment and his or her active and voluntary participation in obtaining a therapeutic result [4]. It can be summarised as a relationship between “what the patient does” and “what the patient and doctor have decided after negotiation without imposition” [5]. The patient no longer simply submits to a proposed treatment, but must be able to discuss or even refuse it. This implies that the patient accepts his or her illness and understands the value of the treatments prescribed. The clinical approach to adherence must be tailored to each individual and must be approached in a holistic, multidimensional manner [6]. A recent systematic review of the literature showed that the identified barriers to adherence in general are as follows: patients’ beliefs about treatment or prioritisation of treatment based on beliefs, patient experiences and abilities, the doctor-patient relationship, health literacy, treatment complexity and social and family support [7]. Another qualitative focus group study focused on the views of chronically ill patients and identified other barriers such as perception of illness, expectation of treatment outcomes, patient autonomy and patients’ perceptions of disease control [8]. Adherence to oral anticancer drugs is a relatively recent phenomenon. This advance has helped to improve the quality of life of patients by reducing side effects and the number of hospital stays [9] compared to traditional chemotherapy. Unlike intravenous chemotherapy where the patient is brought to the hospital for administration, it is more difficult for the prescriber to monitor adherence to treatment, although it is important for its effectiveness. Indeed, the consequences can be measured in terms of loss of immediate or long-term benefits: recurrence or worsening of symptoms, increase in the number of re-hospitalizations [10].

In digestive oncology, non-adherence to capecitabine (the most commonly used oral anticancer drug) is described in 20–30% of cases [11–14]. Non-adherence has many consequences. For the patient, it can sometimes have short-term benefits, such as the disappearance of adverse effects or the reduction of direct drug costs if the patient does not refill his or her prescriptions anymore or only partially. However, from a medical point of view, the consequences are important: loss of chance, progression of the disease, hospitalisation.

It has been reported that patients’ perceptions of their treatments and their motivations appear to be factors affecting adherence [15]. A few studies have assessed variables that might predict poor adherence to oral anticancer drugs, notably in prostate cancer [16] or breast cancer [17, 18]. Another study assessed the determinants of drug adherence in patients taking tyrosine kinase inhibitors [19]. To our knowledge, no study has assessed these determinants in digestive oncology. Given the importance of adherence to oral chemotherapy in this field, the aim of this exploratory study was to identify the factors that may influence adherence to medication, in order to take action in current practice to improve patient support.

## Methods

We used a qualitative approach of semi-structured interviews exploring patients’ perceptions, beliefs and representations to assess the determinants of medication adherence [20].

### Sampling

The study took place at the Nantes University Hospital, in the multipurpose medical oncology unit and in a day hospital. We wished to survey a sample of patients similar to those encountered in routine practice, i.e. with different characteristics that could vary the views: age, type of cancer (location), type of treatment and duration of treatment. Inclusion criteria were as follows: patient over 18 years of age, with cancer of digestive location and with at least one prescription of oral anticancer drugs. The recruitment of patients was stopped at data saturation, when two successive interviews did not lead to the identification of new themes relevant to our research question [21]. Each patient gave consent before starting the interview. Ethical approval was obtained from the Groupe Nantais d’Ethique dans le Domaine de la Santé on 10 September 2020.

### Interviews/data collection

The patient’s referring oncologist introduced the study to the patient by phone. If the patient agreed, the first author contacted the patient to provide further information and to schedule an appointment for a face-to-face, inpatient or post-consultation interview. The interviews were conducted between September 2020 and March 2021, by the first and last author, both of whom were trained in conducting qualitative interviews. The interviews were conducted using a semi-structured interview guide drawn from the literature and with the help of a Doctor in Sociology and qualitative research methodologist. The first part of the guide aimed to explore the patient’s relationship with medication and their health history. The second part explored the patient’s experience of the treatment announcement, their confidence, their fears and the support of those around them. The third part explored more directly the patient’s motivations for adhering to their treatment and their constraints. Finally, the fourth part allowed for reflective feedback and a synthesis of the interview. The interviews were recorded (audio) and then transcribed using the NVIVO V.11 software (QRS International Pty. Ltd., Doncaster, Victoria, Australia).

### Data analysis

After checking the transcripts, the data was analysed independently by the first and fifth author using a thematic approach inspired by Grounded theory [22, 23]. This approach leads to consider data collection and data analysis as simultaneous stages by means of a constant back and forth between the field and the interpretation. Thus, the 4 stages of this analysis (immersion in the data, coding, creation of categories and identification of themes) were conducted by the two authors who compared their analysis until they reached a common interpretation of the verbatims collected. Those interpretations allowed to apprehend events’ sense (i.e. taking or not taking the drug) and to understand the different explicative schemes (i.e. the important of professional activities) until the phenomenon of adherence seems to be fully understood by the authors.

NVIVO V.11 software (QRS International Pty. Ltd., Doncaster, Victoria, Australia) was used for the coding stage. Triangulation was used to improve the quality of data collection, coding and analysis. Thus, in order to avoid any imposition of subjectivity or over-interpretation of the data, the issues raised by the coding and then the analyses were systematically discussed in multidisciplinary team meetings, including a sociologist.

## Results

### Characteristics of the population

Seventeen patients were included in the study. The average interview duration was 30 [12–50] min. The characteristics of the patients are presented in Table 1.

**Table 1:** General characteristics of the participants

Patient ID	Gender	Age (years)	Type of cancer	Treatment	Combination oral + intravenous treatment	Duration of treatment (months)	Duration of interview (min)
1	Female	35	CRC	Capecitabine + oxaliplatin	Yes	1	20
2	Female	45	CRC	Capecitabine + oxaliplatin	Yes	1	35
3	Male	35	GIST	Imatinib	No	17	23
4	Male	59	HCC	Regorafenib	No	11	25
5	Female	89	CRC	Trifluridine/tipiracil + bevacizumab	Yes	4	25
6	Male	60	CRC	Trifluridine/tipiracil + bevacizumab	Yes	6	50
7	Male	78	CRC	Capecitabine + oxaliplatin	Yes	2	26
8	Male	66	CRC	Trifluridine/tipiracil + bevacizumab	Yes	5	40
9	Male	83	GIST	Imatinib	No	42	20
10	Male	41	GIST	Imatinib	No	24	12
11	Female	50	CRC	Capecitabine	No	4	50
12	Female	52	CRC	Regorafenib	No	7	30
13	Male	79	CRC	Encorafenib + cetuximab	Yes	5	45
14	Male	80	HCC	Sorafenib	No	10	32
15	Male	70	HCC	Sorafenib	No	12	30
16	Male	63	CRC	Capecitabine + bevacizumab	Yes	11	22
17	Female	55	Neuroendocrine pancreatic tumor	Sunitinib	No	9	23

Abbreviations: *CRC* colorectal cancer, *HCC* hepatocellular carcinoma, *GIST* gastrointestinal stromal tumour

Eleven were male. The median age was 60 [35–89] years. Ten patients had colorectal cancer, 3 patients had hepatocellular carcinoma, 3 patients had gastro intestinal stromal tumour (GIST) and 1 patient had neuroendocrine pancreatic tumour. Five patients were treated with capecitabine, 3 with trifluridine/tipiracil and 9 with tyrosine kinase inhibitors. Eight patients were treated with a combination of oral and intravenous anticancer drugs.

### Structural analysis

Five major categories that may influence adherence to oral anticancer drugs were identified by the analysis: demographic and socioeconomic factors, disease-related factors, care system factors, treatment-related factors and factors related to patients’ representations and pathways.

### Demographic and socio-economic factors

With regard to demographic and socio-economic factors, three themes were identified: professional activity, medical and social family environment and access to treatment.

Three patients mentioned their professional activity. Indeed, one patient reported to us: “well, I don’t work anymore, so I have time to think and organise myself according to that” when talking about his illness and his treatment. Another patient with a professional activity told us about his difficulties on days when he does not work and is away from his usual routine*:* “when I’m working, I know that at breakfast it becomes a little ritual, but as soon as I leave this work context, on holiday, at the weekend and all that, I sometimes forget quite easily”.

Two patients mentioned their family circle working in the medical-social sector. Indeed, one of the patients declared that he followed his wife’s instructions as a nurse: “(…) I have my doctor at home, so I follow his instructions”. Another said: “my wife is a pharmacist (…), she doesn’t forget, if I tend to forget a bit, I have the right to a reminder”. Finally, two patients mentioned access to treatment: “(…) it’s good that I can take them, treat myself without it costing me a penny from my pocket, so there you go”.

### Disease-related factors

Concerning the factors linked to the disease, only the theme of seriousness, mentioned by two patients, was identified in connection with drug adherence: “it’s too serious to do anything with it” and “unfortunately, we know that one cancer in two wins”.

### Care system-related factors

Regarding factors related to the care system, three themes were identified: the patient’s trust in the oncologist, the quality of information given by the oncologist at the initiation of treatment and the quality of care.

Concerning the patient’s trust in the oncologist, 12 patients mentioned it: “I trust them because I think they are people who know their job well. They do everything to help us”.

Ten patients mentioned the quality of the information given at the start of treatment. For some, the information was clear and appropriate: “he presented it to me as a better way of life, it’s more pleasant” or “what’s good is that she first mentioned the fact that my body was suitable for chemotherapy according to the analyses”. For others, the information given was not verified: “the surgeon told me on the last day when I left that normally there would be no treatment”. This patient shared with us her disappointment at receiving treatment when the surgeon had told her after the operation that she would not have any.

The quality of care was mentioned by 7 patients. Some of them expressed their satisfaction: “If I talk about a problem and they take care of it, if there is an answer, I’m fine. If there’s nothing, I say I’ll stop taking it because it hurts me too much. There, I see for my problems, they take care of it” and others told us about the availability of the care staff: “I was very touched, he told me if you have a problem at any time, never hesitate to call me”. The emblematic verbatims concerning demographic and socio-economic factors, factors related to the disease and to the care system are presented in Table 2.

**Table 2:** Emblematic verbatims concerning demographic and socio-economic factors, disease-related factors and care system factors

Categories	Themes (number of quotes)	Selected verbatims
**Demographic and socio-economic factors**	Professional activity (3/17)	*- Well, I don’t work anymore, so I have time to think and organize myself according to that (P7)*
		*- I'm a bit tired of it but it doesn’t matter,* *I’m retired. We do it the next day and that’s it. (P16)*
		*- When I’m working, I know that at breakfast it becomes a little* *ritual but as soon as I leave this work context,* *on holiday at the weekend and all that, I sometimes forget quite easily (P3)*
	Medical and social family environment (2/17)	*- So my wife is a nurse, so I was following, I have my doctor* *at home, so I was follow his instructions (P3)*
		*- My wife is a pharmacist. She’s used to* *distributing medicines and she doesn’t* *forget, if I tend to forget a bit. I have the right to a reminder (P15)*
	Access to treatment (2/17)	*- The first time I went there was the price of medicines,* *I was a bit shocked because every month it’s* *about 1000 euros, I think it’s 1000 euros,* *I had to see the bill for the medicines… so it’s true* *that it’s not nothing. And we say to ourselves that we are in a beautiful country,* *a beautiful system or I’ve been taking these medicines for two,* *one and a half years, so it’s good that I can take them, treat* *myself without it costing me, a penny from my pocket, so there you go (P3)*
**Disease factors**	Gravity (2/17)	*- Unfortunately, we know that one cancer in two wins (P6)*
		*- It’s too serious to do anything with it (P17)*
		*- Oh, I can’t take that lightly. That’s still there now,* *it’s getting better. But I’d say that since* *2015 I've had this, there hasn’t been five minutes* *in the day when I haven't thought about it (P17)*
**Care system factors**	Patient’s trust in the oncologist (12/17)	*- I avoid going on the Internet because there* *is so much, there is so much contradictory information* *that I don’t look at, I for me, I necessarily ask a medical professional (P2)* *- I trust them because I think they are people who know their job well. They do everything to help us (P12)*
		*- If you don’t trust, you buy a gun and* *then put a bullet in your head, it’s no use (P6)*
		*- I say that it is the person in front of you* *who is qualified to advise you better: if you trust him, you* *do what he tells you to do… (P7)*
	Quality of information given by the *oncologist at the initiation of treatment (10/17)*	**Clear and appropriate information**
		*- What’s good is that she first mentioned the* *fact that my body was suitable for chemotherapy* *according to the analyses (P1)*
		*- And Doctor X she’s very good too, she explains* *things well, it’s important, I think (P13)*
		*- Pff bah the leaflet uh, well fortunately doctor* *X had given me some side effects on another sheet,* *we have another sheet. It’s more succinct, but I still read the leaflet. (P11)*
		*- He presented it to me as a better* *way of life, it’s more pleasant (P16)*
		*- So I was given a sheet with all the side effects.* *So we went through them a little bit and then she told* *me that just because we had a sheet of paper with side effects on both* *sides didn’t mean we were going to get them. But they* *are obliged to tell us everything and that too (P11)*
		**Incorrect information**
		*- And the proof, it was supposed to be harmless for* *me and finally, the first treatment,* *the first week, it was rather a technical knockout so I didn’t* *find out more than that (P1)*
		*- the surgeon told me on the last day when* *I left that normally there would be no treatment (P2)*
	Quality of care (7/17)	**Satisfaction**
		*- That’s why I stayed at the Nantes University Hospital,* *because I find a lot of benevolence and good care.* *They always listen to me, that’s it (P5)*
		*- I think we are part of a team in these cases.* *There’s the doctor, and there’s the patient and these are not* *trivial illnesses, so we’re part of a team (P6)*
		*- If I talk about a problem and they take care of it,* *if there is an answer, I’m fine. If there’s nothing,* *I say I’ll stop taking it because it hurts me too much. There, I see for my problems, they take care of it (P8)*
		*- I am well accompanied by all the staff* *of the University Hospital. I would like to point this out.* *From the first day I came to the emergency room until today (P11)*
		**Availability of care staff**
		*- But knowing, having a phone number, having a name, it’s reassuring,* *you’re not alone. When you take oral chemo, well, you’re all alone at home.* *We take our little tablets and everything,* *and knowing that there’s someone there is important (P11)*
		*- I was very touched, he told me if you have a problem* *at any time, never hesitate to call me (P14)*
		*- My GP who opened the door for me, who said if you* *really have any questions, concerns, you call me (P2)*

### Treatment-related factors

Regarding treatment-related factors, three themes emerged: tolerance of treatment, simplicity of treatment and difficulties with treatment.

Fourteen patients mentioned the tolerance of the treatment. Most patients mentioned toxicity and adverse effects induced by the treatment: palpebral oedema, digestive disorders, hand-foot syndrome, and alopecia. This was the theme most often mentioned by the patients interviewed.

Twelve patients mentioned the simplicity of the treatment, often compared to intravenous treatment: “(…) it’s still less restrictive to swallow tablets than if I had to come twice, three times a week” or “a capsule that I used to combine with a glass of water, I don’t know, there’s more misery. There is more physical interaction that can lead to a feeling of pain. One patient mentioned a beneficial gain in autonomy for the psychic part of the adherence process: “You do your treatment yourself. I already think that on a mental level it is better” and others mentioned a feeling of freedom: “if I move, I can take the pack of medicines with me”.

Finally, 7 patients mentioned difficulties or constraints in following this treatment, such as the fear of forgetting, the large size of the tablets and the way in which they are taken: “waiting 30 min can be a constraint”. The emblematic verbatims concerning factors related to the treatment are presented in Table 3.

**Table 3:** Emblematic verbatims concerning treatment-related factors

Categories	Themes (number of quotes)	Selected verbatims
**Treatment factors**	Tolerance of traitment (14/17)	*- I feel it’s weighing on my eyes and it’s a pain in the ass in the workplace* *because when I get to work it doesn’t look like I’m awake, although I* *am but it makes my face look a bit worse (P3)*
		*- If you like, I’m so sore in both feet that I can’t do the activities* *I used to do because while I was still* *sick, I used to ride an electric bike (laughs) and walk,* *I used to do things to maintain my body. Now I’m reduced to the sofa* *and the remote control. And that’s starting to wear me out… (P8)*
		*- I suffer from it as something a bit disabling.* *Compared to the expectations I have of living a life of* *my age without too many problems, but I think that these drugs,* *this particular drug, was creating a number of problems for me (P14)*
		*- I started losing my hair and eyelashes. And that was dramatic.* *In fact, I didn’t recognize myself.* *That’s what I told the psychologist. It was shocking because what disturbed me* *the most was not recognizing myself* *because it made my eyes bulge and it completely depressed me.* *I said, but it's not possible, it’s not me (P17)*
	Simplicity of treatment (12/17)	***Autonomy***
		*- You do your treatment yourself. I already think* *that on a mental level it is better (P4)*
		***Comparison of intravenous chemotherapies***
		*- If it has the same effect as the infusion,* *then I prefer to swallow four tablets* *in the morning and evening (P2)*
		*- Well, the advantage is that, if I tell you,* *it’s to be at home or to be in one’s* *environment. it’s still less restrictive to swallow tablets* *than if I had to come twice, three times a week (P2)*
		*- The day before, before coming to the appointment, I explained* *to the person I was with, it’s crazy,* *it would be great if this thing could be made into a medicine to be swallowed,* *because with the medicine that we swallow compared to many other things in the medical system.* *It seems … It’s a simple and painless procedure (P1)*
		*- A capsule that I used to combine with a glass of water,* *I don’t know, there’s more misery.* *There is more physical interaction that can lead to a feeling of pain (P1)*
		**Freedom**
		*- If I move, I can take the* *medicine pack with me (P1)*
		*- Well, the fact that it’s under medication and* *not an injection. It’s more, it’s practical, you can travel with* *these drugs (P10)*
		*- Today, in my everyday life, I don’t* *feel like I'm taking a treatment, I do sport if I feel like it.* *I go to parties with friends. I work completely, normally (P3)*
	Difficulties with treatment (7/17)	**Fear of forgetting**
		*- It’s more like forgetting it,* *but without doing it on purpose, that is to* *say that, little by little, as the three weeks go by, you* *feel that you’re coming back to yourself in normal mode.* *So, it’s more a case of forgetting, or at least I’m not a person who is used* *to taking medication. You really have to be assiduous because* *if it’s someone who’s absent-minded …* *it’s not going to work (P1)*
		**Galenic**
		*- When I saw the size at the beginning, I said to myself …* *then you really have to take the time to swallow.* *Because for the first time, I wanted to swallow everything in one* *go, one after the other, and then I had a bit* *of trouble getting through (P2)*
		*- I say to myself, well, they’re quite big and it’s* *all very simple, in general, it follows a medical* *course, we’ve been through quite a lot and the apprehension of* *thinking if I swallow it wrong, well, it’s big and it’s stupid,* *but we’re still quite anxious about the slightest little thing* *and the fact that it’s big isn’t… (P1)*
		**How to take**
		*- Waiting 30 min can be a constraint (P2)*

### Patients’ representations and pathways-related factors

Regarding the factors of adherence related to the patients’ representations and pathways, many themes were identified: representations of medicines, internal resources, external resources, previous experiences with medicines, emotions felt, perceived benefits of treatment and help with the taking.

Five patients reported to us on the representations they had of medicines in general. Some of them had rather positive representations, based on previous experiences, and others rather negative: “it is something unnatural for me, worked, chemical”.

The theme of internal resources was mentioned by 6 patients. We identified 3 sub-themes which are the commitment made at the time of initiation of treatment*:* “you really have to commit yourself to something, it’s not a joke (…)”, the fluctuation of motivation evoked as a “fed up” with the routine and the side effects and finally self-esteem with the notion of fighting against the disease by fighting.

Seven patients mentioned their external resources. Several patients mentioned their family and social environment as playing a beneficial role in medication adherence, whilst others spontaneously mentioned negative beliefs about medication: “but I’m not a fan of using medication. And I have a wife who encourages me not to be a fanatic at all and who would rather I was organic than take the things, you know in alternative medicine”. Some patients also reported using alternative and complementary medicines or supportive care such as meditation, breathing sessions, acupuncture and consultations with a psychologist or magnetist.

Six patients mentioned previous experiences of family and friends with medication, or personal experiences: “a medication that obviously, it seems, was not very, very good and caused me heart problems”.

Eleven patients told us about their emotions when they started the treatment: relief for some, seen as a favourable alternative to treatment with intravenous chemotherapy, but fears and anxieties for others, often linked to the side effects mentioned and the loss of quality of life: “it was not even the fact of swallowing the drugs that posed a problem for me, it was the side effects and I was afraid of losing my hair” or “(…) it bothered me a lot at the beginning to know whether I would be able to continue to do sport tomorrow, to how to put it, evenings out with friends, aperitifs, alcohol and all that, so there were a lot of questions, a lot of apprehension at the beginning before starting this medication”. One patient spoke of shame: “at the beginning I didn’t talk about it much because right away people were like: oh poor guy, oh all that”.

The perception of the benefits of the treatment by the patient was mentioned by 8 patients: “(…) the tumour has halved, so I think it was the right drug and I did well to take it”.

Finally, 6 patients told us about techniques to help them take their medication: timer, annotation on the back of the pack. Emblematic verbatims concerning patient-related factors are presented in Table 4.

**Table 4:** Emblematic verbatims concerning factors related to patients’ representations and pathways

Categories	Themes (number of quotes)	Selected verbatims
**Patient’s representations and pathways factors**	Representation of medicines (5/17)	*- It would be wrong of me to criticize medicines as they have saved me more than once (P8)*
		*- It is something unnatural for me, worked, chemical (P1)*
		*- Knowing that every drug is said to cure you* *on the one hand, and destroy you on the other (P8)*
	Internal resources (6/17)	**Commitment**
		*- After they give me a treatment, I do that,* *I am assiduous and I am stupid and disciplined, they give me,* *I do that (P2)*
		*- You really have to commit to something and it’s not a joke, it’s not* *I take it and then I don’t take it because I don’t* *want to take it, no, it’s I take it and I don’t* *even think about it and I know I have to take it and that’s it.* *There is a protocol to follow and I do it (P2)*
		*- I never gave myself the choice to say to myself: do I take* *it or not? That's how it was, I had to take it (P3)*
		*- I can’t see myself not obeying this kind of treatment.* *I don’t understand why people can’t take it seriously (P17)*
		**Fluctuation of motivation**
		*- We know that fighting and confidence is 50% or* *more than that. If you don’t have that, you don’t have a chance* *for drugs (P6)*
		*- Well, I’ve reached such a state if you like … at the moment,* *it’s pissing me off, but if it’s a stage to get through,* *so be it (P8)*
		*- I’m fed up, maybe just fed up, fed up with the routine* *and the suffering I think. There were times when I had strong* *side effects (P6)*
		**Self-esteem**
		*- I trust the doctor, I trust myself, so I continue to fight and the* *straight line has been drawn, so let’s go (P6)*
		*- I think that even before the disease, at the moment,* *I have the character to fight, so I fight, and I don't go* *looking (P6)*
	External resources (7/17)	**Complementary alternative medicine, supportive care**
		*- What I do, I tell you, is more meditations,* *relaxation, things like that, but more for the mind (P2)*
		*- I am being followed by a psychologist.* *Because what happened to me was a lot. It* *was violent, brutal, so I needed to understand the reasons why.* *So I see her every two weeks at the moment (P11)*
		*- I am very suspicious now of these alternative medicines actually (P17)*
		**Social ressources**
		*- We go for it and I’m well supported by* *my family too. My family plays a role too (P6)*
		**Negative belief in medication by family and friends**
		*- But I'm not a fan of using medication.* *And I have a wife who encourages me not to be a fanatic* *at all and who would rather I was organic than* *take the things, you know in alternative medicine (P14)*
		*- On my father’s side, they are people who (laughs) well,* *they prefer to treat themselves with plants … Well,* *they take medication like me, well if they have a treatment to follow,* *they have to, but they are a bit, well, they are more reticent than me (P2)*
	Previous experiences with medicines (6/17)	**Bad personal experiences**
		*- A medication that obviously, it seems, was not very,* *very good and caused me heart problems (P14)*
		*- I developed a side effect apparently* *that was not necessarily well known (P17)*
		**Experiences of the entourage**
		*- My mum has diabetes and other pathologies.* *I’ve seen her swallow a lot of medicine since* *I was little and that… it’s like a rejection but it’s true that it’s always shocked me (P2)*
		*- Another thing that made me decide to accept it was,* *I thought about my mum: she has no treatment.* *We can’t offer her anything. And me, if they offered me something,* *I couldn’t see myself uh … (P11)*
		*- I know someone who also has the same problem as me 5 years ago.* *And she is doing very well (P11)*
	Emotions felt (11/17)	**Relief**
		*- I was relieved of the medication and oral part because when you* *hear the treatment you may have to deal with, these are words that remain* *quite strong, heavy, traumatic or scary (P1)*
		*- So I was relieved that he might have a solution, an alternative to chemo,* *and I was satisfied that he continued because* *if I didn’t want to do chemo, because I didn’t want to do it,* *I didn’t want to do it anymore because it had been horrible (P8)*
		*- It was a relief to know that it was in pills (P2)*
		**Fears/anxiety**
		*- It was not even the fact of swallowing the drugs that posed a problem for me, it was the side effects and I was afraid of losing my hair (P2)*
		*- At first, it’s a bit scary when you see all the possible side effects.* *It’s true that it makes you think ‘oh’ (P12)*
		*- The day before I was told about it or the day before,* *everything was fine, so, um, it was also a question of knowing* *whether it would change my daily life. it bothered me a lot at the beginning to* *know whether I would be able to continue to do sport tomorrow,* *to how to put it, evenings out with friends, aperitifs, alcohol and all that,* *so there were a lot of questions, a lot of apprehension at the* *beginning before starting this medication (P3)*
		*- The apprehension was mainly, um, I didn’t want* *my daily life to be disrupted, so not too much.* *And work too, because work was important to me, so um …* *I heard that there were other people who were off work.* *It’s clearly something I didn’t want (P3)*
		*- I was starting to feel apprehensive and so much so that I said to myself,* *‘Gosh, what have I got myself into? Yes, why did I agree to what. And then, yeah,* *at the beginning of each cycle, the day before,* *I’m not in the mood (P11)*
		*- I’m actually afraid of the side effects.* *Much more than whether it works or not. I’m afraid of the side effects that might diminish my quality of life actually, that might prevent me from living normally (P17)*
		**Shame/guilt**
		*- At the beginning I didn’t talk about it much because* *right away people were like: oh poor guy, oh all that (P3)*
		*- They’re afraid, it’s a big word right away, tumors,* *cancer, people, especially at my age,* *it’s grandma who’s 80 years’ old who talks about it, well we say* *to ourselves that’s nature, that’s how it is (laughs).* *I’m a bit younger, so people tend to be a bit … I can see that it’s* *hard for others and I don’t want people to feel sorry for me,* *so that’s why, especially as I don’t need it (P3)*
	Perceived benefits of treatment (8/17)	*- I was saying to Dr X earlier, what’s harder* *for me is that I don’t see the purpose (P11)*
		*- That said, I recognize that he also does things* *that are good for me. We had the results just now, it’s not* *just anything (P14)*
		*- There is no improvement but there is no deterioration, so the treatment* *we give you stabilizes the disease well (P16)*
		*- The important thing is to see if it works and the* *tumor has halved, so I think it was the right drug and I did* *well to take it (P3)*
	Help with the taking (6/17)	*- I set a 30-min timer in the middle of the meal,* *or at the end of the meal (P7)*
		*- I put a timer on anyway, because you have to take them* *30 min after the meal. I have a timer, so I look at the* *time to say to myself, well, at such and such a time, because it’s certain* *that you can quickly go and do something else and* *then you forget, that’s why I say to you, you* *really have to be square*
		*- I write down the initials of the days of* *the week on the back of the plate so that I can remember* *whether I have taken it or not (P3)*

## Discussion

The aim of this study was to identify the factors that influence the process of adherence to oral anticancer drugs in patients with digestive cancer, in order to better understand it and to act in practice to improve patient support. We identified 5 categories of factors: demographic and socio-economic factors, factors related to the disease, factors related to the care system, factors related to the treatment and factors related to the patient’s representations and pathway.

With regard to demographic and socio-economic factors, we note that the absence of professional activity seems to be favourable to adherence. This is what we find in the literature [24]. It therefore seems essential to take an interest in the organisation of a patient with a professional activity, by asking them to describe a typical day and by encouraging, for example, a treatment schedule compatible with their routine to improve adherence.

Regarding factors related to the health care system, the availability of health care staff was a recurring theme. The availability of the nursing staff is a positive factor found in the literature that contributes to the therapeutic alliance and promotes satisfaction with the patient’s care [25–27]. One patient told us about her loneliness: “(…) when you take oral chemo, well, you are all alone at home. We take our little tablets and everything (…)”. It is in this situation that the availability of the nursing staff becomes very important for the patient. The same patient reported: “but to know, to have a telephone number, to have a name, it’s reassuring, you’re not alone (…) and to know that there’s someone, it’s important”, referring to the nurse coordinator of the service who ensures a personalised follow-up [28, 29] to alleviate the patient’s difficulties and loneliness. Hence, the importance, once again, of knowing the patient’s environment, listening to them and understanding them in order to qualify their needs. The same applies to the quality of the patient-physician relationship, which has been widely described as a key to the adherence process [30–32]. Indeed, patients seek to have complete confidence in their oncologist and wish to be informed and reassured in order to perceive his or her expertise. More than two-thirds of the patients surveyed mentioned the importance of trusting their oncologist. It has been reported that information given at the beginning of treatment is one of the factors of good compliance and helps to avoid treatment errors [33], provided that the information given is adapted to the patients’ needs. However, the link between understanding/knowledge of the treatment and compliance has not been systematically demonstrated [34]: this knowledge may give rise to fears, whilst ignorance may be a defence strategy for some patients. It is at this point in the treatment process that a pharmaceutical interview can be offered to the patient to encourage adherence. The aim of these interviews is to support the patient in the follow-up of their treatment so that they understand it, take ownership of it and adhere to it through an exchange of views and an active listening posture in order to provide them with information adapted to their needs, taking into account their stage of acceptance of the disease and their level of literacy.

Regarding treatment-related factors, the toxicity of oral anticancer drugs mentioned by almost all the patients interviewed is a negative factor in adherence [35, 36]. This poor tolerance can affect two of the three phases of the adherence process: implementation, if the patient does not take the oral treatment voluntarily, or persistence, if the patient stops the treatment earlier than expected. Visible side effects such as hair loss or palpebral oedema are very difficult for patients to experience. Toxicities that reduce the quality of life, such as hand-foot syndrome that handicaps the patient’s autonomy, or significant digestive disorders, are frequently cited by the patients we met. It is at this point in the management process that supportive care and complementary alternative medicine make sense to alleviate these toxicities and therefore the difficulties in implementing or continuing them are crucial [37]. A patient may be referred to a therapeutic education programme to acquire self-care skills for a better quality of life or to a medical specialist depending on their need.

However, one patient sees the side effects as the price to pay for the effectiveness of the treatment: “you have to fight against the side effects and fight them”. This perceived link between efficacy and side effects can have negative consequences. On the other hand, the simplicity of the treatment appeared to be an asset for its adherence. The patient gains autonomy and freedom to accept the treatment.

Concerning factors related to patients’ representations and pathways, several patients told us of their fears at the beginning of the treatment. These elements support the idea of combining a pharmaceutical initiation consultation with the medical consultation in order to understand the patient’s fears about the treatment and to reassure him/her on this subject using motivational support tools [38]. The fact that the patient perceives the caregiver’s confidence in their ability to manage their medication encourages them to adhere to the treatment [39]. The representations of medicines mentioned by the patients interviewed are widely described as a barrier to adherence [40, 41], especially when they are reinforced by the beliefs and representations of the patient’s entourage. It is essential to highlight these at the start of treatment. The patient’s previous experience with drugs will also play a role in the adherence process [42], especially when the patient has had a bad experience with an adverse reaction [43]. On the other hand, the perception of the therapeutic benefit of the treatment is a favourable factor [44]. It is therefore important to take the time to explain to the patient that the treatment works when it does so that he understands the benefit of their action in adhering to their treatment: the patient believes that he can control the events through their actions [45]. One of the advantages of these pharmaceutical interviews could be to offer patients, when necessary, an aid to taking the medication: timer, annotation on the back of the pack, telephone alarm. In the digital age, other means not mentioned by the patients interviewed could have a significant impact on improving compliance [46, 47].

### Limits

Our study has limitations. This study has been thought as exploratory; thus, the field was limited to one hospital, the one in where the authors are employed. Moreover, the profile of the respondents is extremely diverse (age, type of cancer, treatment received and duration of treatment, socioeconomic characteristics…). The patient perceptions presented in this study cannot cover the full range of views of patients with digestive cancer prescribed an oral anticancer drug. Those elements limit the generalisability of the results, even if data saturation seems to have been reached [48]. The results presented here are hypothesis which should help to design actions in order to improve patient’ support.

Secondly, a majority of patients had previously received intravenous chemotherapy. This experience seems to have influenced their views on the oral anticancer drug. Thirdly, we found very few themes related to the disease. This imbalance compared to the other categories is probably explained by the way our interview guide was written, mainly focused on the drug itself.

## Conclusion

The present study identifies areas for improvement to increase adherence to oral anticancer drugs in patients with digestive cancer, such as proposing pharmaceutical discussions at the start of treatment, or referring patients to a therapeutic education programme. The results of this study could help healthcare professionals to adapt their practices according to the problems of adherence encountered. This study demonstrates the value of observing these determinants as a whole. Adherence to medication is an individual construct that requires interaction with the patient in order to identify the elements that influence their behaviour and to find levers and a strategy for action accordingly.

## Supplementary Information

Below is the link to the electronic supplementary material.

Supplementary file1 (PDF 241 KB)

## Data Availability

NA.

## Code availability

NA.
