# Supplementary material for: Exploring the factors influencing adherence to oral anticancer drugs in patients with digestive cancer: a qualitative study
Source: Support Care Cancer. 2021 Nov 23;30(3):2591–604. doi: 10.1007/s00520-021-06663-2 (PMC8794904; doi:10.1007/s00520-021-06663-2)
Supplement: Supplementary file 1 — Supplementary file1 (PDF 241 KB) [file 520_2021_6663_MOESM1_ESM.pdf]

Interview guide

- What did you think of drugs before your cancer?
    - Do you regularly take medication for chronic problems?
        - Which ones?
        - Why?
    - Do you ever take medicines without a prescription? That you buy yourself?
        - Which ones?
        - Why?
        - Where do you find the information? Pharmacist, doctor, Internet ?
        - How do you choose?
    - Do you discuss prescriptions with your doctor?
    - How would you describe your health knowledge?

- What did you think when it was suggested that you switch to oral anticancer drugs?
    - How did it go?
        - Who suggested it to you?
        - What have you been told?
        - With what words?
        - What did you say?
        - What did you experience?
        - Remember what came immediately to mind.
    - Did you know about this type of treatment?
    - Did the doctor give you any information/explanations?
        - Did you need more information?
        - Who did you discuss it with?
    - What happened between the proposal and the acceptance of the treatment?
    - Have you consulted anyone else? Doctors, relatives, internet?
    - Do you know anyone who has had cancer?
    - What kind of treatment did he receive?
    - How did it go?

- How are you taking your treatment or how has it been since you started taking the oral treatment?
    - If you have been thinking about not taking it, do you remember what came to your mind in that thought?
        - What constraints did you perceive?
        - What benefits did you perceive?
    - Do you now regret taking it?
    - What is "taking your medication well"?
    - What is the current status of your treatments?
    - Do you do anything else to fight the disease?

- If you knew someone like you, what would you say?
    - How would you present your medicine to him?
    - What suggestions would you have for improvement?
- Have I forgotten something? Do you want to discuss something around this?
